# Metformin activates type I interferon signaling against HCV via activation of adenosine monophosphate-activated protein kinase

**DOI:** 10.18632/oncotarget.20248

**Published:** 2017-08-14

**Authors:** Wei-Lun Tsai, Tsung-Hsien Chang, Wei-Chi Sun, Hoi-Hung Chan, Chun-Ching Wu, Ping-I Hsu, Jin-Shiung Cheng, Ming-Lung Yu

**Affiliations:** ^1^ Division of Gastroenterology and Hepatology, Department of Internal Medicine, Kaohsiung Veterans General Hospital, Kaohsiung, Taiwan; ^2^ School of Medicine, National Yang-Ming University, Taipei, Taiwan; ^3^ Department of Medical Education and Research, Kaohsiung Veterans General Hospital, Kaohsiung, Taiwan; ^4^ Department of Medical Laboratory Science and Biotechnology, Chung Hwa University of Medical Technology, Tainan, Taiwan; ^5^ Hepatobiliary Division, Department of Internal Medicine and Hepatitis Center, Kaohsiung Medical University Hospital, Kaohsiung, Taiwan; ^6^ Faculty of Internal Medicine, College of Medicine, and Graduate Institute of Clinical Medicine, and Lipid Science and Aging Research Center, Kaohsiung Medical University, Kaohsiung, Taiwan; ^7^ Institute of Biomedical Sciences, National Sun Yat-Sen University, Kaohsiung, Taiwan

**Keywords:** metformin, hepatits C virus, interferon, AMPK

## Abstract

Activation of the type I interferon (IFN) signaling pathway is essential for the eradication of hepatitis C virus (HCV). Metformin can activate adenosine monophosphate-activated protein kinase (AMPK) to reduce insulin resistance. Cross talks between AMPK and IFN signaling remain unclear. To understand the influence of metformin on the type I IFN signaling pathway and HCV infection, the full-length HCV replicon OR6 cells and the infectious HCV clones JFH1 were used to assess the anti-HCV effect of the insulin sensitizers, metformin and pioglitazone. Immunofluorescence staining and the immunoblotting of HCV viral protein demonstrated that metformin, but not pioglitazone, inhibited HCV replication in OR-6 and JFH-1-infected Huh 7.5.1 cells. Immunoblotting data showed that metformin activated the phosphorylation of STAT-1 and STAT-2 in OR-6 and JFH-1 infected Huh 7.5.1 cells. Metformin enhanced the phosphorylation of AMPK, and the metformin-activated IFN signaling was down-regulated by AMPK inhibitor. After treatment of AMPK inhibitor, the level of HCV core protein decreased by metformin can be rescued. In conclusion, metformin activates type I interferon signaling and inhibits the replication of HCV via activation of AMPK.

## INTRODUCTION

Hepatitis C virus (HCV) infection is a major cause of liver cirrhosis and hepatocellular carcinoma (HCC) in Taiwan [1]. In patients with acute HCV infection, 60-90% will develop a chronic infection and after 20-30 years of infection, 20-30% will develop cirrhosis of the liver or hepatocellular carcinoma [2, 3]. HCV causes many metabolic problems besides its damage of liver pathology. Among them, insulin resistance (IR) is a very important one [4]. HCV proteins can influence the functions of mitochondria and endoplasmic reticulum, increase reactive oxygen species (ROS) production, activate P-38 mitogen activate protein kinase (MAPK) and nuclear factor -kappa B (NF-κB), affect the expression of many cytokines such as TNF-alpha, -beta, interleukin 6 and 8, and inhibit insulin receptor substrate (IRS) as well as down-regulating adiponectin and causing IR [5, 6]. IR is associated with fatty liver and causes rapid progression of liver fibrosis [5–8] as well as being associated with poor response to interferon (IFN) and ribavirin combination therapy [9, 10].

Metformin and pioglitazone are the two major insulin sensitizers in clinical practice. Metformin is an oral biguonide, which activates AMP-activated protein kinase (AMPK) to inhibit the output of glucose from liver; and increases the utility of peripheral glucose to reduce insulin resistance [11–14]. Metformin, pegylated IFNα-2a and ribavirin combination therapy has a trend to increase the sustained virological response (SVR) rate in chronic HCV patients and actually increase the SVR rate in female patients [15]. Pioglitazone is a thiazolidinedione, which reduces IR in the liver and peripheral tissues by stimulating the nuclear receptors peroxisome proliferator-activated receptor (PPAR)-γ and -α that control the expression of insulin-sensitive genes [16, 17]. Treatment with pioglitazone before and during treatment with pegylated IFNα-2a plus ribavirin improves glycemic control in patients with chronic hepatitis C and insulin resistance, but this regiment does not enhance the virological response [18].

Activation of the IFN signaling pathway is an important mechanism against HCV infection. IFN-α has been used in HCV treatment for over two decades. Type I IFNs exert their potent antiviral effects through regulating hundreds of IFN-stimulated genes (ISGs) [19, 20]. Type I IFNs bind to the cell surface receptor (IFNAR) and activate the receptor-associated tyrosine kinases Jak1 and Tyk2. The kinases then phosphorylate and activate STAT1 and STAT2. Phosphorylated STAT-1 and STAT-2 recruit a third factor, IRF9, to form a complex known as IFN-stimulated gene factor 3 (ISGF3), which translocates into the nucleus, binds to IFN-stimulated response elements (ISRE) and induces the transcription of ISGs, many of which are thought to confer anti-HCV effects [21–23].

The influence of metformin on the type I interferon signaling pathway has never been explored before. Besides, the interplay between AMPK and the type I interferon signaling pathway remains unclear. Thus, we analyzed the role of metformin in HCV replication, and its influences in type I interferon signaling pathway. In addition, the effect of AMPK inhibition in metformin-regulated type I IFN signaling was investigated.

## RESULTS

### Effect of metformin on HCV replication in a full-length replicon, OR6 cells

**Figure 1:**
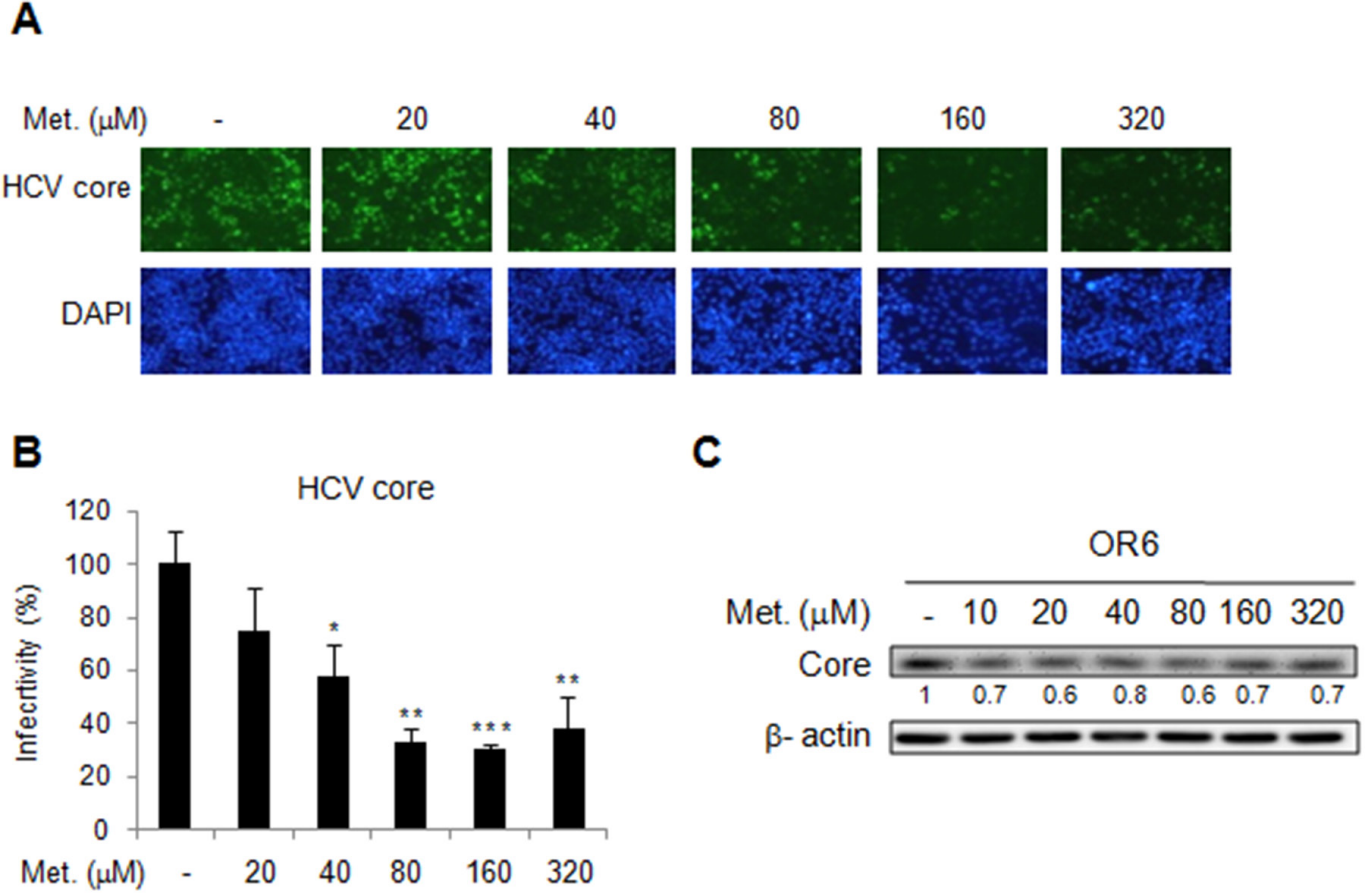
Effects of metformin on HCV replication in OR-6 cells HCV core protein expression was determined in OR-6 cells treated with different doses of metformin for 48 h **(A)** Immunofluorescence assay (IFA) with anti-HCV core antibody (upper panels) and DAPI staining of nuclei (lower panels) were shown. **(B)** Quantification of IFA was performed. ^*^P < 0.05, ^**^P < 0.01, ^***^P < 0.001 in metformin treated versus untreated control group. **(C)** The cell lysates from OR-6 cells treated with different doses of metformin for 48 h, were analyzed by immunoblotting with anti-HCV core protein antibody, and the β-actin was shown as the loading control.

The OR-6 cells were treated with different doses of metformin for 48 h. Significant decrease of HCV core protein expression after 48 h of metformin treatment was observed in immunofluorescence assay(Figure 1A and 1B). Immunoblotting also showed metformin reduced the levels of HCV core protein in the full-length OR6 replicon cells (Figure 1C). Collectively, our results indicated that metformin exerts an antiviral effect against HCV replication in the replicon system.

### Effect of metformin on HCV replication in infectious JFH1 cell line

**Figure 2:**
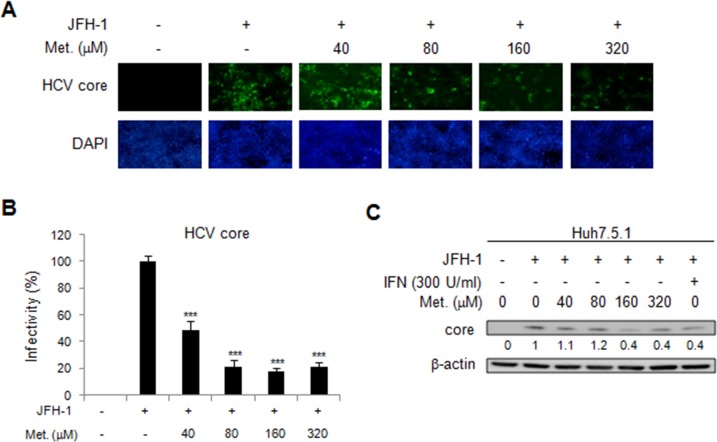
Effects of metformin on HCV replication in JFH-1 infected Huh 7.5.1 cells The Huh7.5.1 cells were infected by JFH1 for 72 h and then treated with different doses of metformin for 48 h. **(A)** Immunofluorescence assay using anti-HCV core antibody was performed. IFA of anti-HCV core antibody (upper panels) and DAPI staining of nuclei (lower panels) were shown. **(B)** Quantification of IFA was performed. ^*^P < 0.05, ^**^P < 0.01, ^***^P < 0.001 in metformin treated versus untreated control group. **(C)** JFH-1 infected Huh 7.5.1 cells were treated with different doses of metformin for 48 h and the cell lysates were analyzed by immunoblotting with anti-HCV core protein antibody.

We then assessed the effect of metformin on HCV replication in another genotype of HCV, JFH1 (genotype 2a) with a viral infection cell model. The Huh7.5.1 cells were infected by JFH1 for 72 h and then treated with different doses of metformin for 48 h. Immunofluorescence assay found the significantly decreased signals of HCV core protein in metformin treated cells (Figure 2A and 2B). Moreover, metformin treatment reduced the level of HCV core protein expression in JFH-1-infected Huh 7.5.1 cells in immunoblotting assay (Figure 2C). These data demonstrated the anti-HCV activity of metformin in JFH-1-infected Huh7.5.1 cells.

### Effect of pioglitazone on HCV replication in HCV replicon and infectious clone systems

**Figure 3:**
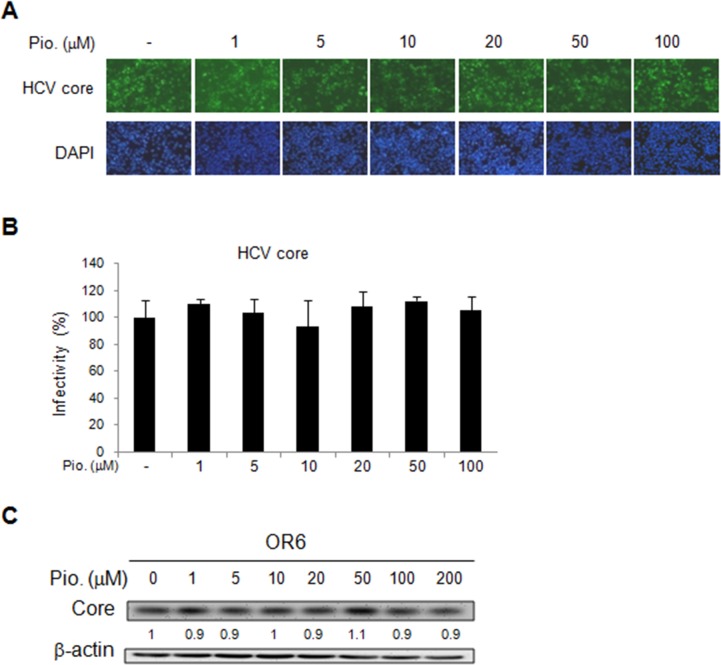
Effects of pioglitazone on HCV replication in OR-6 cells HCV core protein expression was determined in OR-6 cells treated with different doses of pioglitazone for 48 h. **(A)** Immunofluorescence assay (IFA) of anti-HCV core antibody (upper panels) and DAPI staining of nuclei (lower panels) were shown. **(B)** Quantification of IFA was performed and no significant difference between pioglitazone treated and untreated control group was found. **(C)** The cell lysates harvested from OR-6 cells treated with different doses of pioglitazone and were analyzed by immunoblotting with anti-HCV core protein antibody.

**Figure 4:**
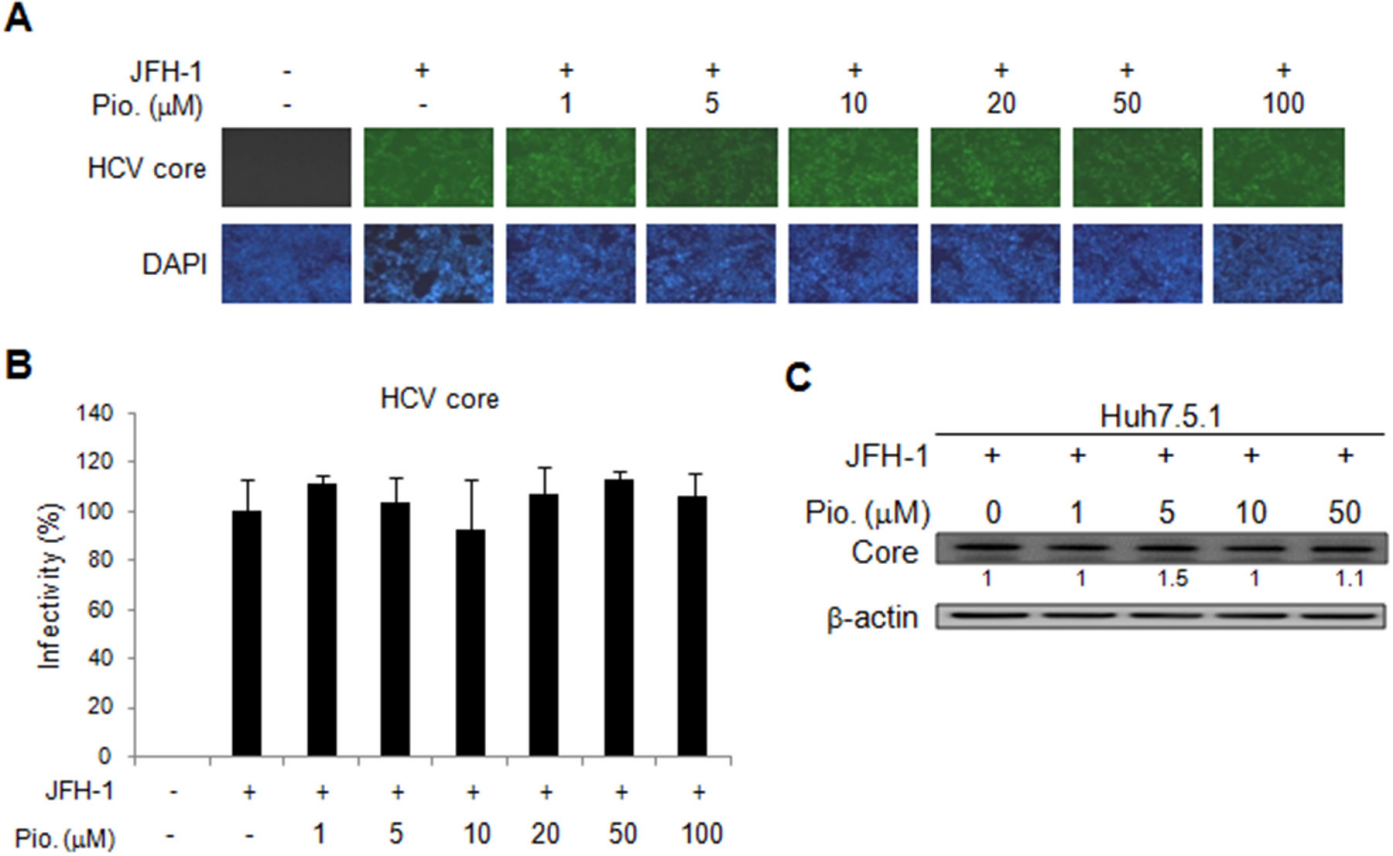
Effects of pioglitazone on HCV replication in JFH-1 infected Huh 7.5.1 cells The Huh7.5.1 cells were infected by JFH1 for 72 h and then treated with different doses of pioglitazone for 48 h. **(A)** Immunofluorescence assay using anti-HCV core antibody was performed. IFA of anti-HCV core antibody (upper panels) and DAPI staining of nuclei (lower panels) were shown. **(B)** Quantification of IFA was performed and no significant difference between pioglitazone treated and untreated control group was found. **(C)** JFH-1 infected Huh 7.5.1 cells were treated with different doses of pioglitazone for 48 h, then and cell lysates were analyzed by immunoblotting with anti-HCV core protein antibody.

We further evaluated effect of another insulin sensitizer, pioglitazone, on HCV replication by using the OR6 cells treated with different dosage of pioglitazone for 48 h. Unlike metformin, pioglitazone treatment did not influence HCV replication, which was demonstrated by the immunofluorescence assay for HCV core protein (Figure 3A and 3B) as well as immunoblotting of HCV core protein(Figure 3C). Similar results were shown in JFH-1- infected Huh7.1 cells. The expressions of core protein were not affected by pioglitazone (Figure 4A-4C). These results indicated that pioglitazone had no effect on HCV replication in cell model.

The cytotoxicity of metformin and pioglitazone were evaluated in Huh7.5.1 cells. The results showed that the cell viability was not impaired by metformin treatment (40 μM – 640 μM), thus the metformin inhibited HCV replication was not due to the cytotoxic effect (Supplementary Figure 1A). However, high dose of pioglitazone (100 μM) induced cell death was determined (Supplementary Figure 1B).

### Metformin activates the type I IFN antiviral signaling pathway

**Figure 5:**
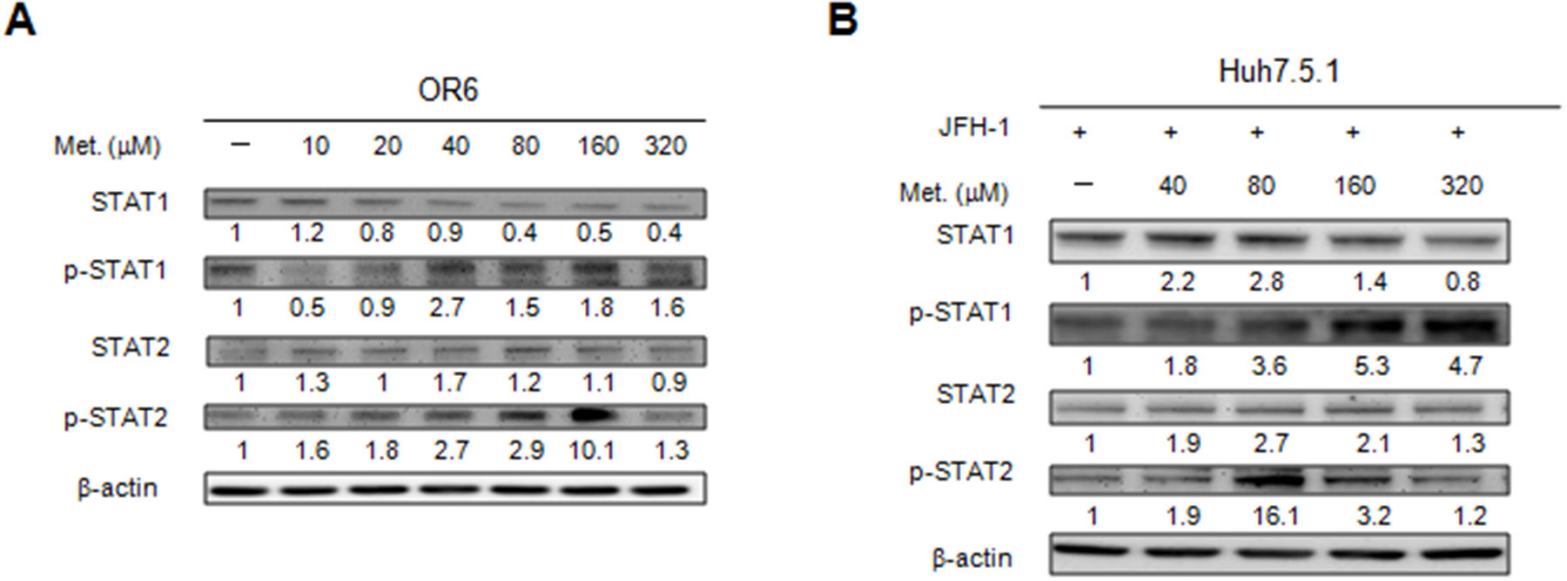
Effects of metformin on type I IFN signaling pathway The cell extracts were harvested from OR6 cells **(A)** or JFH1-infected Huh7.5.1 cells **(B)** with or without metformin (10∼320 μM) treatment for 48 h, the expression of IFN signaling proteins were analyzed by immunoblotting with the specific antibodies.

To understand whether metformin could regulate the type I IFN-mediated antiviral signaling; we determined the signaling proteins by using immunoblotting analysis in metformin-treated OR6 and JFH1-infected Huh7.5.1 cells. We found that metformin increased STAT1 and STAT2 expression and also induced their phosphorylation in OR6 cells (Figure 5A) and JFH-1-infected cells (Figure 5B). Metformin also activated STAT2 phosphorylation in the uninfected Huh7.5.1 cells, (Supplementary Figure 2). These data demonstrated that the metformin activated JAK–STAT signaling pathway of host antiviral machinery.

### Metformin activates type I IFN signaling pathway in an AMPK dependent manner

**Figure 6:**
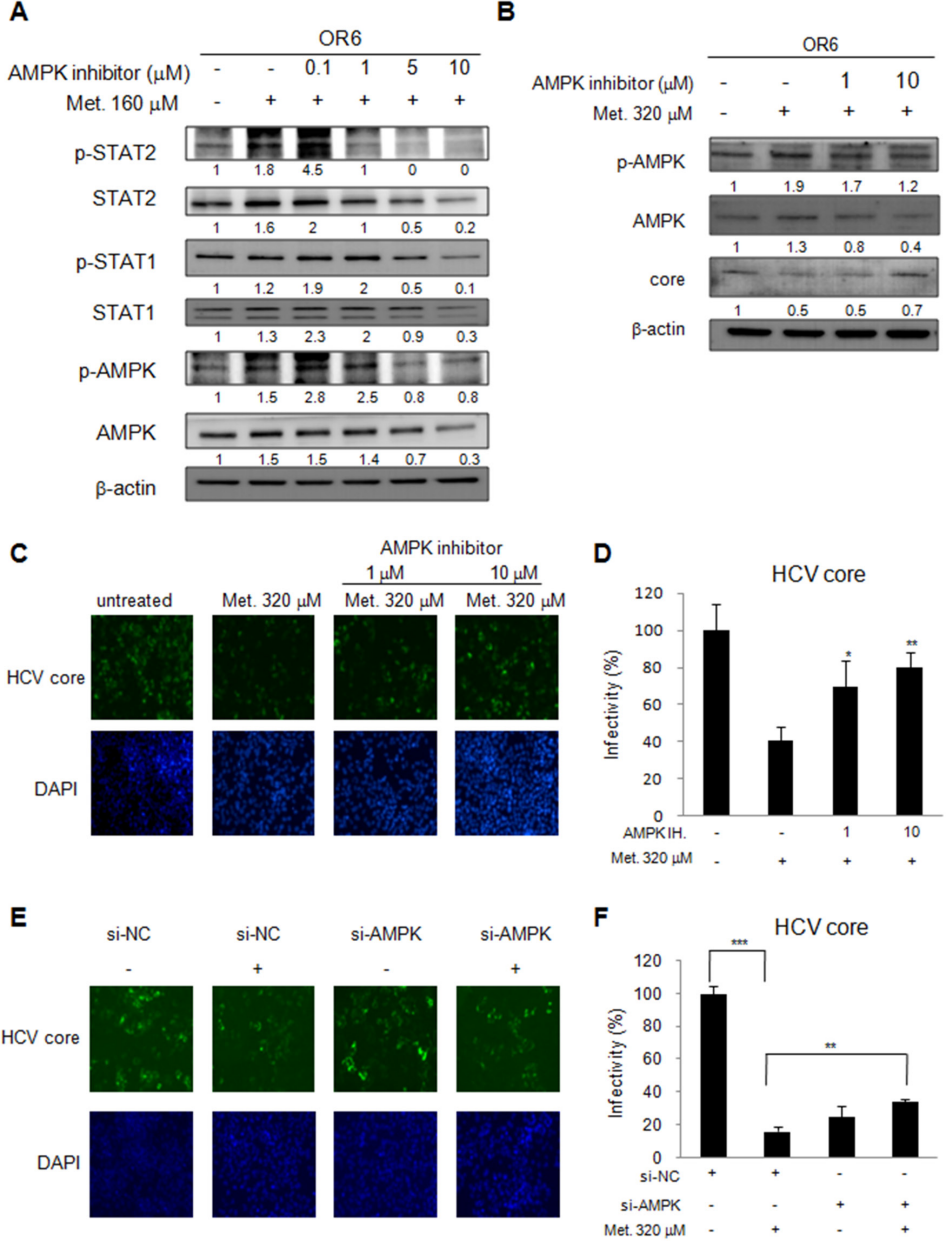
Metformin activates IFN signaling pathway in an AMPK dependent manner **(A)** OR6 cells were treated with AMPK inhibitor (0.1∼10 μM) for 30 min then stimulated with metformin (160 μM) for 48 hrs. The whole cell extracts were analyzed by immunoblotting with indicated antibodies. **(B)** OR6 cells were treated with AMPK inhibitor (1 and 10 μM) for 30 min then stimulated with metformin (320 μM) for 6 hrs. The whole cell extracts were analyzed by Immunoblotting with indicated antibodies. **(C)** Immunofluorescence assay using anti-HCV core antibody was performed in OR6 cells treated with AMPK inhibitor (1 and 10 μM) for 30 min then stimulated with metformin (320 μM) for 6 hrs. IFA of anti-HCV core antibody (upper panels) and DAPI staining of nuclei (lower panels) were shown. **(D)** Quantification of IFA was performed. ^*^P < 0.05, ^**^P < 0.01 in AMPK inhibitor and metformin treated group versus metformin alone treated group. **(E)** Immunofluorescence assay of HCV core protein was conducted in OR6 cells with AMPK siRNA knockdown, DAPI staining indicates cell nuclei. **(F)** The infectivity of HCV in AMPK knockdown cells was quantified. Student’s t test between groups, ^**^P < 0.01, ^***^P < 0.001

Metformin is an AMPK activator, thus, to determine whether AMPK influences metformin activated IFN signaling, OR6 cells were treated with different dose of AMPK inhibitor (0.1 μM-10 μM) for 30 min then followed by metformin (160 μM) treatment for 48 h. Immunoblotting showed that AMPK phosphorylation was activated in OR6 cells with metformin treatment (Figure 6A). However, the metformin-mediated STAT1 and STAT2 phosphorylation was downregulated by treating with AMPK inhibitor in a dose-dependent manner (Figure 6A). Our data suggested that metformin activates the type I IFN anti-viral signaling pathway via activation of AMPK.

We further evaluated the effect of AMPK inhibitor on metformin-induced HCV suppression. OR6 cells were treated with AMPK inhibitor (1 and 10 μM) for 30 min then stimulated with metformin (320 μM) for 6 hrs. Immunoblotting (Figure 6B) and immunofluorescence assay (Figure 6C and 6D) showed that the decreased expression of HCV core protein by metformin in OR6 cells was reversed when co-administered with AMPK inhibitor. The siRNA knockdown strategy was applied in AMPK inhibition. After knockdown of AMPK with siRNA transfection, the reduction of HCV core protein by metformin was significantly rescued (Figure 6E and 6F).

Interestingly, we found that the efficacy of type I IFN against HCV was enhanced by metformin treatment in OR6 cells (Supplementary Figure 4A-4C). These data suggest a combination of IFN plus metformin would be a possible regimen for HCV treatment in clinical practices.

## DISCUSSION

Metformin is the major insulin sensitizer for the treatment of diabetes. In this study, we found metformin activated type I interferon signaling pathway and inhibited HCV replication via activation of AMPK.

Previous studies found adding pioglitazone to an IFN-α and ribavirin combination regimen failed to improve the SVR rate [18], despite the improvement in IR. However, in female patients treated with metformin, IFN-α and ribavirin, SVR rate was increased along with the improvement in IR [15]. These clinical studies leave two unsolved questions; first, insulin sensitivity is improved in both studies, but why did the pioglitazone included arm fail to improve the treatment outcome; second, what is the mechanism underlying the improved outcome by metformin. Although metformin and pioglitazine can both effectively improve insulin sensitivity, they worked on by different mechanisms, which might therefore have different effects on HCV replication. In this study we found metformin, but not pioglitazone, has inhibitory effect on HCV replication. This may partly explain the different treatment outcomes with either of the two drugs when combined with IFN-based therapy for chronic HCV infection.

IFN-α activates JAK/STAT signaling pathway and induces the expression of hundreds of ISGs encoding innate immune effectors that influence the control of HCV replication [21, 24, 25]. The effects of metformin on IFN signaling pathway remain largely unclear. Our study found metformin-inhibited replication of HCV was associated with IFN signaling activation in hepatoma cells harboring genotype 2a (JFH-1 infected Huh 7.5.1 cells) and genotype 1b (OR-6 cells). Metformin restricted the replication of HCV of different genotypes and in the era of directly acting antiviral agents for the treatment of HCV [26, 27], metformin may play another role in the viewpoint of immunoregulation. However, further clinical studies are required to prove its effects.

Several studies have demonstrated the anti-HCV effects of metformin, however, non of the studies had explored the activation of innate immunity by metformin against HCV replication. Goto et al. in a recent study discovered that SNARK kinase inhibitor metformin suppressed both HCV replication and SNARK-mediated enhancement of TGF-β signaling [28]. Huang et al. demonstrated that HC V inhibited the AKT-TSC -MTORC1 pathway via ER stress, resulting in autophagy, which may contribute to the establishment of the HCV-induced autophagy [29]. Campo et al. in a recent study reported that HCV replication was inhibited *in vitro* by metformin [30]. Nakashima et al., in a recent study found that inhibition of HCV replication through AMPK- dependent and -independent pathways [31]. Another recent study by Mankouri et al. found that inhibition of AMPK is required for HCV replication [32]. Our study should be the first report to demonstrate the activation of innate immunity against HCV by metformin.

AMPK is one of the central regulators of cellular and organismal metabolism in eukaryotes [33, 34]. AMPK also has critical roles in the regulation of growth and reprogramming metabolism [13, 14]. Metformin impairs ATP production, activating the conserved sensor of nutritional stress AMPK, thus providing a plausible and generally accepted model for suppression of gluconeogenic gene expression and glucose output [13]. The cross talks between IFN and AMPK signal pathway remained unclear. More recently, AMPK was proposed to be involved in immunity to viruses [31, 35]. However the precise mechanism underlying STAT phosphorylation by AMPK remained unclear. Prantner et al. in a recent study found that loss of AMPK led to dephosphorylation at Ser 555 of the Modulation of Stimulator of Interferon Genes (STING) regulator, UNC-51-like kinase 1 (ULK1) and ULK1-deficient cells responded normally to DMXAA, indicating that AMPK promotes STING- dependent signaling independent of ULK1 in mouse cells and AMPK promotes Innate Immunity and antiviral defense through modulation of STING signaling [36]. Besides, another study also found that ULK1 activation occurred following disassociation from its repressor AMPK, and was elicited by cyclic di nucleotides (CDN)'s generated by the cGAMP synthase, cGAS. Thus, while CDN's may initially facilitate STING function, they subsequently trigger negative-feedback control of STING activity, thus preventing the persistent transcription of innate immune genes [37]. In the current study, we firstly reported that metformin activated the type I IFN signaling, which could be suppressed by AMPK inhibitor and the restrictive effect of metformin on HCV can also be rescued by AMPK inhibitor.

Although DAAs are very effective in the treatment of HCV infection, in some special populations such as decompensated cirrhosis or patients with multi-drug resistant RASs, DAAs are still not ideal. Metformin is a very safe drug and widely used for the treatment of diabetes. We here have demonstrated that metformin is able to inhibit the replication of HCV through their effects of activation of innate immunity, so metformin may possible play some roles in the treatment of HCV when combined with DAAs. But the effects of the combination of metformin with DAAs for the treatment of HCV requires further study to clarify.

In conclusion, our results demonstrated that metformin inhibits HCV replication by enhanced type I IFN antiviral pathway through the activation of AMPK.

## MATERIALS AND METHODS

### Cells, virus and reagents

Huh7.5.1 cells were grown in Dulbecco's Modified Eagle's Medium (DMEM) supplemented with 10% fetal bovine serum (FBS). The infectious JFH1 plasmid was obtained from Dr. Takaji Wakita and inoculated as previously described [38]. The OR6 cell line was obtained from Dr. Nobuyuki Kato [39], which harbors full-length genotype 1b HCV RNA and then grown in DMEM supplemented with 10% FBS and 500 μg/ml of G418 (Promega, Madison, WI). AMPK inhibitor was purchased from EMD Chemicals, Inc. (Gibbstown, NJ).

### Immunofluorescence assay

Immunofluorescence staining of HCV core protein in OR6 cells and JFH1-infected Huh7.5.1 cells were performed as previously described [38]. OR6, Huh7.5.1 or JFH1 cells were fixed with 4% paraformaldehyde, permeabilized with 0.5% TritonX-100, and blocked with 3% bovine serum albumin in PBS. The primary antibody was mouse anti-HCV core (Thermoscientific). The secondary antibody was goat anti–mouse Alexa Fluor 488 (Invitrogen). DAPI was added to the staining to monitor the nuclear structure. Fluorescence signals were observed by fluorescence microscopy (Zeiss, Axcio Observer A1).

### Immunoblotting assay

Cells were lysed using radioimmune precipitation assay (RIPA) buffer containing 1% NP-40, 0.1% SDS, 10 mM Tris–HCl (pH 7.4), 1 mM EDTA, 150 mM NaCl and protease inhibitor cocktail (Roche), and sonification was performed subsequently. Proteins were separated by SDS–PAGE or NuPAGE Novex pre-cast 4–12% Bis–Tris gradient gels (Invitrogen, Carlsbad, CA) and transferred to PVDF membranes. The primary antibodies used were: anti-STAT1, anti-phospho-STAT1 (Tyr701) anti-STAT2 and anti-phospho-STAT2 (Cell Signaling Technology, Inc., Beverly, MA), mouse anti-HCV core, (Thermoscientific), and mouse anti-actin (Sigma Life Science and Biochemicals, St. Louis, MO). Secondary antibodies were: HRP-conjugated ECL donkey anti-rabbit IgG and HRP-conjugated ECL sheep anti-mouse IgG (Amersham Biosciences, Piscataway, NJ). The ECL Western Blotting Detection Kit (Amersham Biosciences, Piscataway, NJ) was used to detect the chemiluminescent signals.

### SiRNA and transfection

SMART pool siRNA for AMPK for gene knockdown were purchased from Dharmacon. The negative control siRNA was obtained from QIAGEN. Lipofectamine^™^ RNAiMAX Transfection Reagent (Invitrogen, Carlsbad, CA) was used for siRNA transfection. Protein expression of the knockdowned gene was confirmed by immunoblotting.

### Statistical analysis

Quantitative data are presented as mean ± standard deviation. Student's t test was used to determine the significance between treatment groups. P < 0.05 was considered statistically significant.

## SUPPLEMENTARY MATERIALS FIGURES



## References

[1] Kao JH, Chen DS (2005). Changing disease burden of hepatocellular carcinoma in the Far East and Southeast Asia. Liver Int.

[2] Santantonio T, Wiegand J, Gerlach JT (2008). Acute hepatitis C: current status and remaining challenges. J Hepatol.

[3] Fattovich G, Stroffolini T, Zagni I, Donato F (2004). Hepatocellular carcinoma in cirrhosis: incidence and risk factors. Gastroenterology.

[4] Shintani Y, Fujie H, Miyoshi H, Tsutsumi T, Tsukamoto K, Kimura S, Moriya K, Koike K (2004). Hepatitis C virus infection and diabetes: direct involvement of the virus in the development of insulin resistance. Gastroenterology.

[5] Sheikh MY, Choi J, Qadri I, Friedman JE, Sanyal AJ (2008). Hepatitis C virus infection: molecular pathways to metabolic syndrome. Hepatology.

[6] Tardif KD, Waris G, Siddiqui A (2005). Hepatitis C virus, ER stress, and oxidative stress. Trends Microbiol.

[7] Hui JM, Sud A, Farrell GC, Bandara P, Byth K, Kench JG, McCaughan GW, George J (2003). Insulin resistance is associated with chronic hepatitis C virus infection and fibrosis progression [corrected]. Gastroenterology.

[8] Moucari R, Asselah T, Cazals-Hatem D, Voitot H, Boyer N, Ripault MP, Sobesky R, Martinot-Peignoux M, Maylin S, Nicolas-Chanoine MH, Paradis V, Vidaud M, Valla D (2008). Insulin resistance in chronic hepatitis C: association with genotypes 1 and 4, serum HCV RNA level, and liver fibrosis. Gastroenterology.

[9] Chu CJ, Lee SD, Hung TH, Lin HC, Hwang SJ, Lee FY, Lu RH, Yu MI, Chang CY, Yang PL, Lee CY, Chang FY (2009). Insulin resistance is a major determinant of sustained virological response in genotype 1 chronic hepatitis C patients receiving peginterferon alpha-2b plus ribavirin. Aliment Pharmacol Ther.

[10] Dai CY, Huang JF, Hsieh MY, Hou NJ, Lin ZY, Chen SC, Hsieh MY, Wang LY, Chang WY, Chuang WL, Yu ML (2009). Insulin resistance predicts response to peginterferon-alpha/ribavirin combination therapy in chronic hepatitis C patients. J Hepatol.

[11] Kahn BB, Alquier T, Carling D, Hardie DG (2005). AMP-activated protein kinase: ancient energy gauge provides clues to modern understanding of metabolism. Cell Metab.

[12] Zhou G, Myers R, Li Y, Chen Y, Shen X, Fenyk-Melody J, Wu M, Ventre J, Doebber T, Fujii N, Musi N, Hirshman MF, Goodyear LJ, Moller DE (2001). Role of AMP-activated protein kinase in mechanism of metformin action. J Clin Invest.

[13] Miller RA, Birnbaum MJ (2010). An energetic tale of AMPK-independent effects of metformin. J Clin Invest.

[14] Towler MC, Hardie DG (2007). AMP-activated protein kinase in metabolic control and insulin signaling. Circ Res.

[15] Romero-Gomez M, Diago M, Andrade RJ, Calleja JL, Salmeron J, Fernández-Rodríguez CM, Solà R, García-Samaniego J, Herrerías JM, De la Mata M, Moreno-Otero R, Nuñez O, Olveira A (2009). Treatment of insulin resistance with metformin in naive genotype 1 chronic hepatitis C patients receiving peginterferon alfa-2a plus ribavirin. Hepatology.

[16] Mayerson AB, Hundal RS, Dufour S, Lebon V, Befroy D, Cline GW, Enocksson S, Inzucchi SE, Shulman GI, Petersen KF (2002). The effects of rosiglitazone on insulin sensitivity, lipolysis, and hepatic and skeletal muscle triglyceride content in patients with type 2 diabetes. Diabetes.

[17] Yki-Jarvinen H (2004). Thiazolidinediones. N Engl J Med.

[18] Harrison SA, Hamzeh FM, Han J, Pandya PK, Sheikh MY, Vierling JM (2012). Chronic hepatitis C genotype 1 patients with insulin resistance treated with pioglitazone and peginterferon alpha-2a plus ribavirin. Hepatology.

[19] Zhao H, Lin W, Kumthip K, Cheng D, Fusco DN, Hofmann O, Jilg N, Tai AW, Goto K, Zhang L, Hide W, Jang JY, Peng LF, Chung RT (2012). A functional genomic screen reveals novel host genes that mediate interferon-alpha's effects against hepatitis C virus. J Hepatol.

[20] Der SD, Zhou A, Williams BR, Silverman RH (1998). Identification of genes differentially regulated by interferon alpha, beta, or gamma using oligonucleotide arrays. Proc Natl Acad Sci U S A.

[21] Darnell JE, Kerr IM, Stark GR (1994). Jak-STAT pathways and transcriptional activation in response to IFNs and other extracellular signaling proteins. Science.

[22] Schindler C, Plumlee C (2008). Inteferons pen the JAK-STAT pathway. Semin Cell Dev Biol.

[23] Liu SY, Sanchez DJ, Aliyari R, Lu S, Cheng G (2012). Systematic identification of type I and type II interferon-induced antiviral factors. Proc Natl Acad Sci U S A.

[24] Horner SM, Gale M (2013). Regulation of hepatic innate immunity by hepatitis C virus. Nat Med.

[25] Metz P, Reuter A, Bender S, Bartenschlager R (2013). Interferon-stimulated genes and their role in controlling hepatitis C virus. J Hepatol.

[26] deLemos AS, Chung RT (2014). Hepatitis C treatment: an incipient therapeutic revolution. Trends Mol Med.

[27] Pawlotsky JM (2014). New hepatitis C therapies: the toolbox, strategies, and challenges. Gastroenterology.

[28] Goto K, Lin W, Zhang L, Jilg N, Shao RX, Schaefer EA, Zhao H, Fusco DN, Peng LF, Kato N, Chung RT (2013). The AMPK-related kinase SNARK regulates hepatitis C virus replication and pathogenesis through enhancement of TGF-β signaling. J Hepatol.

[29] Huang H, Kang R, Wang J, Luo G, Yang W, Zhao Z (2013). Hepatitis C virus inhibits AKT-tuberous sclerosis complex (TSC), the mechanistic target of rapamycin (MTOR) pathway, through endoplasmic reticulum stress to induce autophagy. Autophagy.

[30] del Campo JA, García-Valdecasas M, Rojas L, Á Rojas, Romero-Gómez M (2012). The hepatitis C virus modulates insulin signaling pathway in vitro promoting insulin resistance. PLoS One.

[31] Nakashima K, Takeuchi K, Chihara K, Hotta H, Sada K (2011). Inhibition of hepatitis C virus replication through adenosine monophosphate-activated protein kinase-dependent and -independent pathways. Microbiol Immunol.

[32] Mankouri J, Tedbury PR, Gretton S, Hughes ME, Griffin SD, Dallas ML, Green KA, Hardie DG, Peers C, Harris M (2010). Enhanced hepatitis C virus genome replication and lipid accumulation mediated by inhibition of AMP-activated protein kinase. Proc Natl Acad Sci U S A.

[33] Mihaylova MM, Shaw RJ (2011). The AMPK signalling pathway coordinates cell growth, autophagy and metabolism. Nat Cell Biol.

[34] Steinberg GR, Kemp BE (2009). AMPK in health and disease. Physiol Rev.

[35] Moser TS, Schieffer D, Cherry S (2012). AMP-activated kinase restricts Rift Valley fever virus infection by inhibiting fatty acid synthesis. PLoS Pathog.

[36] Prantner D, Perkins DJ, Vogel SN (2017). AMP-activated kinase (AMPK) promotes innate immunity and antiviral defense through modulation of stimulator of interferon genes (STING) signaling. J Biol Chem.

[37] Konno H, Konno K, Barber GN (2013). Cyclic dinucleotides trigger ULK1 (ATG1) phosphorylation of STING to prevent sustained innate immune signaling. Cell.

[38] Lin W, Tsai WL, Shao RX, Wu G, Peng LF, Barlow LL, Chung WJ, Zhang L, Zhao H, Jang JY, Chung RT (2010). Hepatitis C virus regulates transforming growth factor beta1 production through the generation of reactive oxygen species in a nuclear factor kappaB-dependent manner. Gastroenterology.

[39] Ikeda M, Abe K, Dansako H, Nakamura T, Naka K, Kato N (2005). Efficient replication of a full-length hepatitis C virus genome, strain O, in cell culture, and development of a luciferase reporter system. Biochem Biophys Res Commun.

