# Supplementary material for: Metformin activates type I interferon signaling against HCV via activation of adenosine monophosphate-activated protein kinase
Source: Oncotarget. 2017 Aug 14;8(54):91928–37. doi: 10.18632/oncotarget.20248 (PMC5696152; doi:10.18632/oncotarget.20248)
Supplement: Supplementary file 1 [file oncotarget-08-91928-s001.pdf]

Metformin activates type I interferon signaling against HCV via activation of adenosine monophosphate-activated protein kinase

SUPPLEMENTARY MATERIALS

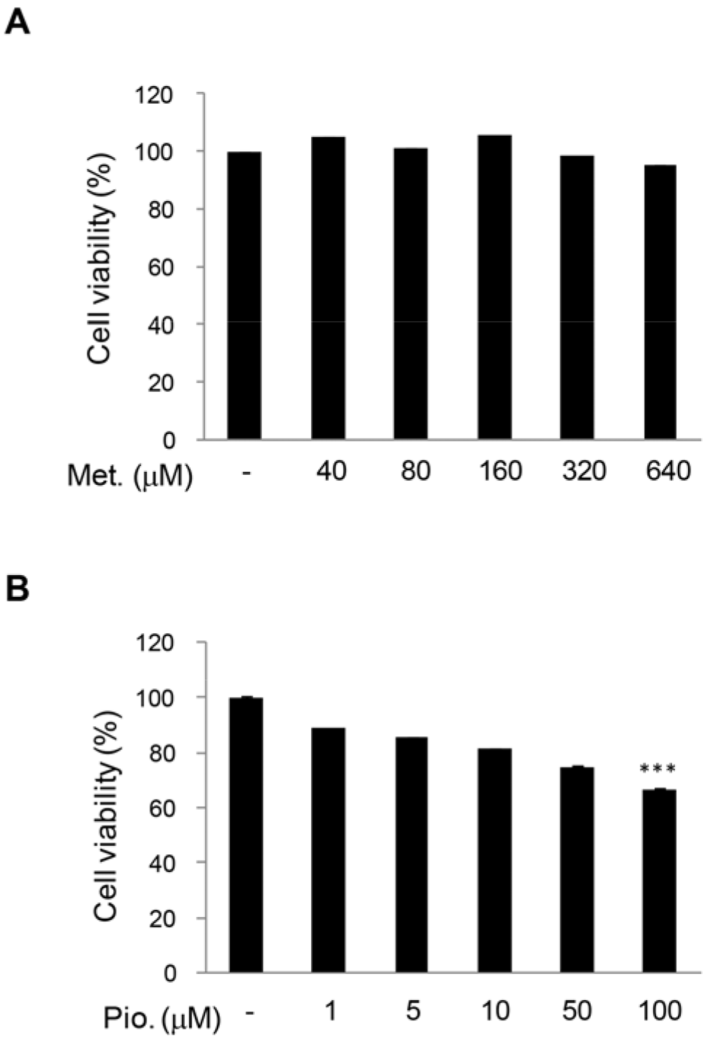

Supplementary Figure 1: The cytotoxicity effect of metformin and pioglitazone. Huh 7.5.1 cells were treated with metformin **(A)** or pioglitazone **(B)** for 48 h and the cell proliferation was determined by WST-1 assay.

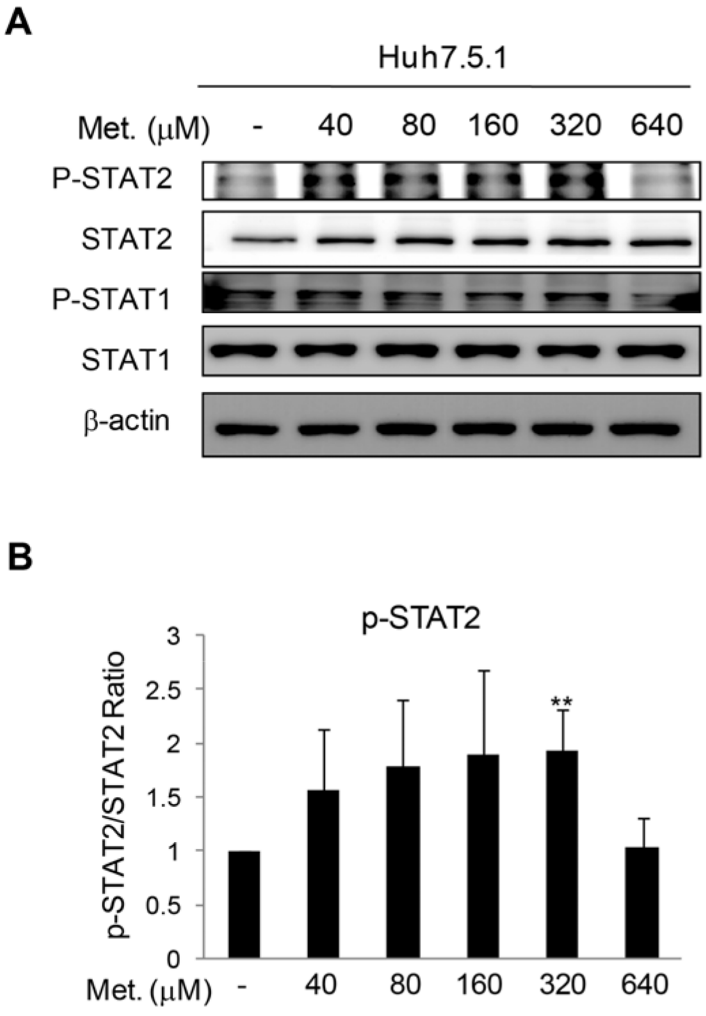

Supplementary Figure 2: The activity of metformin on type I IFN signaling pathway in Huh 7.5.1 cells. (A) The cell lysates from Huh 7.5.1 cells treated with different doses of metformin for 48 h, were analyzed by immunoblotting with anti-HCV core protein antibody, and the β-actin was shown as the loading control. **(B)** Densitometry analysis was performed with ImageJ software. Data are mean ± SD from 3 independent tests. Statistical significance was tested by Student's t-test, *P*-values, **<0.01, in metformin- treated versus the untreated control group.

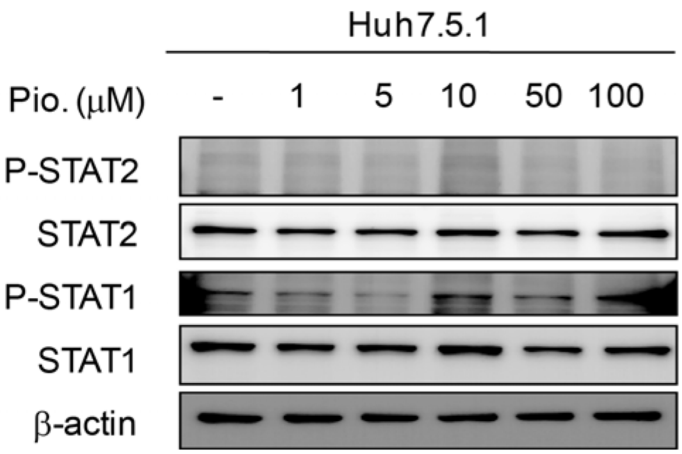

Supplementary Figure 3: The effects of pioglitazone on STAT-1 and STAT-2 phosphorylation. The cell extracts were harvested from Huh 7.5.1 cells with or without metformin (10~320 μM) treatment for 48 h. The expression of IFN signaling proteins were analyzed by immunoblotting with the specific antibodies.

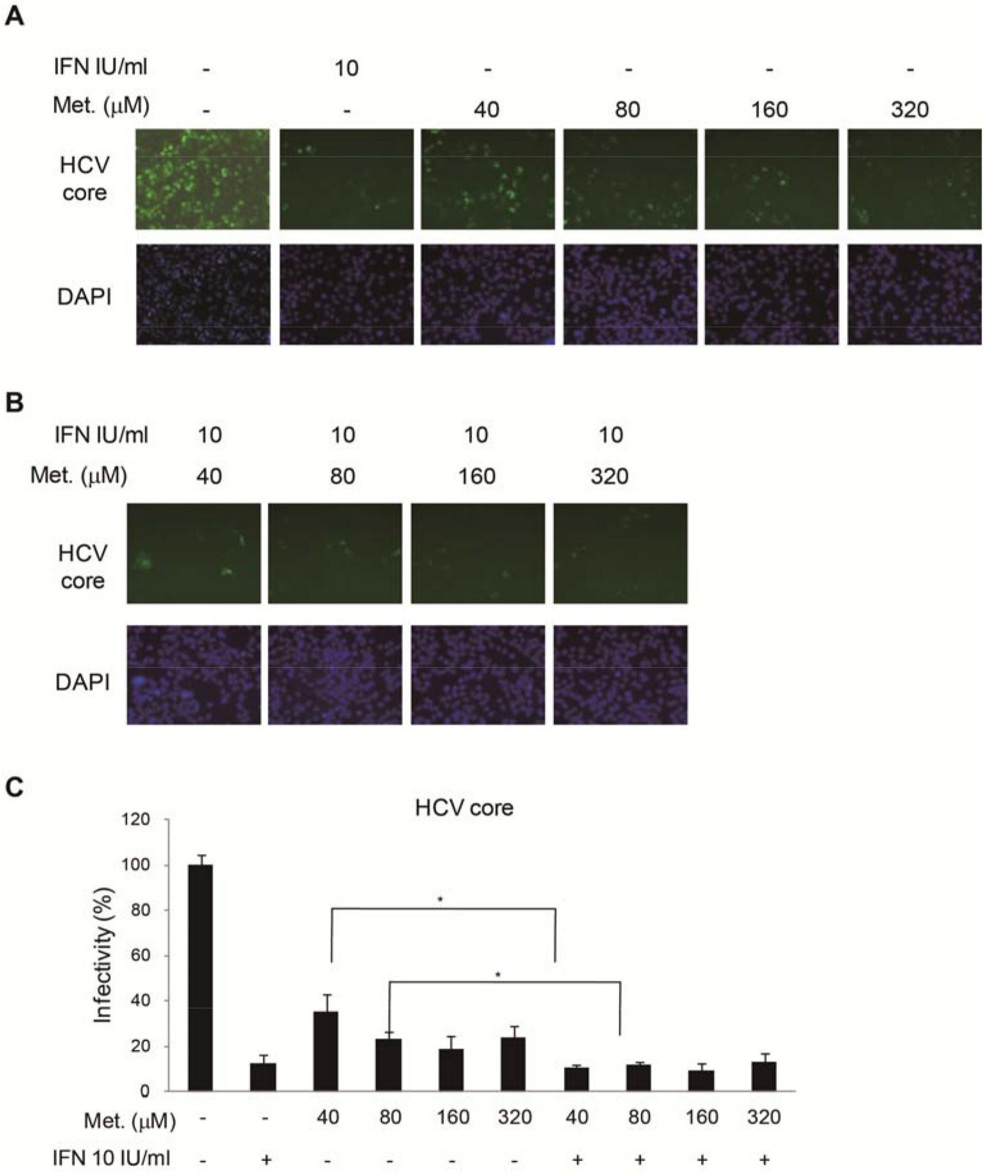

Supplementary Figure 4: The effects of combination treatment with IFN and metformin on the HCV replication. HCV core protein expression was determined by immunofluorescence assay (IFA) of anti-HCV core antibody (upper panels) and DAPI staining of nuclei (lower panels) in OR-6 cells treated with IFN-α or different doses of metformin for 48 h **(A)** or combined treatment of IFN-α and different doses of metformin **(B)**. Quantification of IFA was performed **(C)**. *P < 0.05 in metformin-treated versus combined metformin and IFN-α treatment group.
